# Targeting the ferroptosis pathway for rheumatoid arthritis: molecular mechanisms and prospects for inhibitor development

**DOI:** 10.3389/fimmu.2025.1610121

**Published:** 2025-06-10

**Authors:** Zhuoling Wang, Xinyue Bai, Huahua Zhang, Min Yang, Meilin Liu, Tingyu Nie, Tianjiao Li, Mingru Zhang, Xingdan Wang, Jin Wang, Jiming Han, Xiaolong Liu

**Affiliations:** ^1^ Medical Research and Experiment Center, Yan’an Medical College of Yan’an University, Yan’an, China; ^2^ Nursing Department of Yan’an University Affiliated Hospital, Yan’an, China

**Keywords:** ferroptosis, rheumatoid arthritis, lipid peroxidation, natural medicine, nanomedicine

## Abstract

Rheumatoid arthritis (RA) is a chronic systemic autoimmune disease with severe complications. Ferroptosis, an iron-dependent form of apoptosis, encompasses mechanisms including iron overload, lipid peroxidation, redox homeostasis, and reactive oxygen species accumulation, all of which are closely related to RA pathogenesis. This study focused on the mechanism of ferroptosis and RA, detailing their relationship and outlining the reported roles of ferroptosis inhibitors in RA treatment to provide a useful research basis in drug discovery and development and for clinicians.

## 1 Introduction

Rheumatoid arthritis (RA) is a chronic systemic autoimmune disease (1), initially described by Dr. Landré-Beauvais in 1880 (2). It occurs in individuals aged 30–50 years, especially women, smokers, or those with a familial predisposition (3). RA is typically characterized by synovial joint pain, swelling, and morning stiffness lasting up to 1 h, with initial symptoms usually affecting the hands and feet, particularly the metacarpophalangeal and metatarsophalangeal joints (4). Additionally, RA is characterized by symmetric joint inflammation (5), resulting in damage to articular cartilage and bones, potentially leading to disability (6), and affects approximately 1% of the global population (7), with a two- to three-fold higher prevalence in females than in males (8).The impact on the patient’s quality of life is profound.The pathogenesis mainly involves the interaction of genetic and environmental factors with the immune system, leading to disruption of immune tolerance, autoantibody production, and excessive release of inflammatory cytokines, which in turn leads to chronic synovitis and joint damage.These inflammatory responses are amplified through the activation of multiple signaling pathways (e.g., JAK/STAT, NF-κB, etc.) and cell types (e.g., T-cells, B-cells, synovial fibroblasts, etc.), with the abnormal activation of T-cells leading to an amplified inflammatory response, and the excessive proliferation and activation of synovial fibroblasts directly participating in the inflammation and destruction of joints, which further exacerbates the joint inflammation and tissue destruction (9). Current treatments for RA include nonsteroidal anti-inflammatory drugs (NSAIDs), disease-modifying antirheumatic drugs (DMARDs), biologics, and small-molecule-targeted drugs (JAK inhibitors). NSAIDs relieve symptoms but do not slow RA progression and may cause gastrointestinal adverse reactions with long-term administration. DMARDs, including methotrexate, are the drugs of choice; however, some patients may not tolerate them, and they have poor efficacy. Biologics and targeted drugs are effective in controlling inflammation; however, they are expensive, have the potential risk of infection, and have non-response in some patients (10).

Ferroptosis is an iron-dependent form of apoptosis (11) characterized by iron-dependent lipid peroxidation, a reduction in glutathione peroxidase 4 (GPX4) activity, and reactive oxygen species (ROS)-induced lipid peroxidation (12). Ferroptosis exacerbates the inflammatory response by releasing inflammatory mediators (tumor necrosis factor-alpha [TNF-α], IL-1β, and IL-6). Recently, the role of ferroptosis in RA pathogenesis has been gaining attention. In synoviocytes from patients with RA, RSL3 can induce intracellular lipid peroxidation buildup and subsequently trigger ferroptosis by inhibiting GPX4 activity (13). In addition, ferroptosis inhibitors, including liproxstatin-1, exhibited significant anti-inflammatory benefits in a mouse model of collagen-induced arthritis (CIA) (14), indicating that ferroptosis may be implicated in the pathologic process of RA. Therefore, this review initially introduces the relationship between ferroptosis and RA, examines the mechanism of ferroptosis in RA, assesses its viability as a therapeutic target, outlines existing compounds that mitigate RA by inhibiting ferroptosis, and anticipates future avenues for ferroptosis research in RA, essential for a comprehensive understanding of the pathophysiological process of RA, and for uncovering the potential of targeted inhibition of ferroptosis. A comprehensive understanding of the pathophysiological mechanisms of RA and the development of treatment options for RA aimed at inhibiting ferroptosis hold substantial scientific and therapeutic importance.

## 2 Pathogenesis of RA

### 2.1 Environment and genetics

RA pathogenesis involves the interaction of genetic and environmental factors. Genetic factors include specific alleles of the essential key gene HLA-DRB1, which trigger autoimmune responses by activating T cells through misrecognition of self-antigens, and non-HLA genes, including PTPN22, STAT4, and TRAF1/C5, which affect immune tolerance and the release of inflammatory factors (15). Besides, epigenetic regulation, including aberrant DNA methylation and histone modification, may result in overexpression of pro-inflammatory genes (16). Environmental factors include smoking, which increases the risk of RA by inducing protein citrullination and oxidative stress, infections including Epstein-Barr virus and porphyromonas gingivalis that may trigger autoimmunity through molecular mimicry, dysbiosis of the intestinal flora that could activate toll-like receptors to facilitate Th17 differentiation, and hormonal and metabolic factors including estrogen and obesity that may affect the development of RA. Collectively, these genetic and environmental factors result in the disruption of immune tolerance, autoantibody production, activation of the complement system and inflammatory cells, and, eventually, joint destruction (17).

### 2.2 Abnormalities in the immune system

The primary manifestations of immune system abnormalities are disruption of immunological tolerance and autoantibody production. Disruption of immunological tolerance involves several genes, including specific alleles of the HLA-DRB1 gene HLA-DRB1*04:01) which activate T cells through misrecognition of self-antigens (citrullinated proteins), triggering autoimmune responses (18); the PTPN22 R620W mutation affects T-cell receptor signaling, leading to enhanced T-cell recognition of and response to self-antigens (19); the *STAT4* gene participates in pro-inflammatory cytokine signaling and enhances the activation of Th1 and Th17 cells (20); the *TRAF1/C5* genes are associated with the NF-κB pathway and modulate the release of inflammatory factors (21). Autoantibody generation includes rheumatoid factor (RF) and anti-cyclic citrullinated peptide antibodies (ACPA). RF exacerbates joint inflammation by activating the complement system and inflammatory cells, while ACPA production is closely associated with the “shared epitope” configuration of the *HLA-DRB1* gene, which exacerbates joint inflammation by activating the complement system and inflammatory cells. These immune system anomalies result in the breakdown of immunological tolerance and the generation of autoantibodies, subsequently leading to joint inflammation and destruction (22).

### 2.3 Inflammation and joint damage

In RA, the secretion of inflammatory cytokines, including TNF-α, IL-1β, and IL-6, is implicated in disease progression, and these factors promote the inflammatory response and are directly involved in joint tissue destruction (23). For instance, TNF-α, a pleiotropic cytokine synthesized by many cell types, is essential for the immune cascade response, primarily mediating stress-activated protein kinase/milk mitogen-activated protein kinase (SAPK/MAPK) pathway signaling and activating JAK/STAT signaling, which serves as the major trigger of inflammation and joint damage in RA (24). IL-1β and IL-6 can exacerbate the inflammatory state of joints by increasing the inflammatory response (25). The inflammatory response results in the destruction of articular cartilage and bone, which is the primary cause of disability in patients with RA. Inflammatory cytokines stimulate the activation of synovial fibroblasts and osteoclasts through the activation of multiple signaling pathways, and these cells release matrix metalloproteinases and osteoclast-specific enzymes, leading to the degradation of cartilage and bone. Additionally, synovial fibroblasts proliferate abnormally in the inflammatory environment, forming aggressive cell clusters that exacerbate articular cartilage and bone (26). The persistence of these inflammatory cycles leads to the destruction of bone and cartilage from several angles, rendering RA a disease that cannot be cured by monotherapy (27).

## 3 Mechanisms of ferroptosis

### 3.1 Iron metabolism

Iron is the most abundant and in-demand trace element in the human body (28). All the iron in the human body is derived from the daily diet, and when (Fe^3+^) in food reaches the small intestine, most of it is reduced to (Fe^2+^) by duodenal cytochrome B reductase 1 (duodenal cytochrome b) (29). Subsequently, divalent iron is transferred from the intestinal lumen to the intracellular space through the divalent metal transporter protein 1 (DMT1) (30). The storage and utilization of divalent iron within the cell predominantly rely on transferrin receptor 1 (TfR1) and ferritin, primarily expressed on the cell membrane, and bind to transferrin (Tf) to form the Tf-TfR1 complex, facilitating endocytosis of iron. Ferritin can store ferric ions in its internal iron pool (31). When cells require iron, iron ions are released from ferritin into the iron pool, thereby maintaining the unstable iron pool at low levels and preventing cytotoxicity (32, 33). The released (Fe^2+^) is sensitive to a Fenton reaction catalyzed by O_2_-ions with H_2_O_2_ in the cell, generating ROS, namely hydroxyl radicals, thus inducing ferroptosis in the cell (34).

### 3.2 Lipid peroxidation

Lipid peroxidation is the oxidation of unsaturated fatty acids within cell membranes to generate lipid peroxides, which destroy cell structure and function (35). However, GPX4 mitigates lipid peroxides (including phospholipid hydroperoxides) by converting them into their respective alcohols, thereby inhibiting the chain reaction of lipid peroxidation and protecting the cell membrane from further oxidative damage (36). Additionally, in the Fenton reaction, LOOH (lipid peroxides) react with ferrous ions (Fe^2+^) to generate hydroxyl radicals (-OH), potent oxidants that can damage phospholipids in the cell membrane, especially those abundant in polyunsaturated fatty acids (PUFAs), leading to cell membrane destruction (37). Another feature of lipid peroxidation in Ferroptosis is the autoxidation of esterified PUFA in cell membranes; for instance, arachidonic acid or adenosine deaminase is the most oxidized lipids that are most susceptible to oxidation in Acyl-CoA Synthetase Long-Chain Family Member 4 (ALSL4) and Lysophosphatidylcholine acyltransferase 3 (LPCAT3). LPCAT3 and those form phosphatidylacetamide (lysophosphatidylcholine acyltransferase 3, PES), which is converted to lipid peroxides by lipoxygenase (38).

### 3.3 Imbalance in the antioxidant system

#### 3.3.1 System Xc/GSH/GPX4 axis

The Xc/GSH/GPX4 axis comprises the catalytic subunit (solute carrier family 7 member 11 SLC7A11) and the regulatory subunit (solute carrier family 3 member 2 SLC3A2) interconnected by a disulfide bond. This axis facilitates the retrograde transport of extracellular cysteine and intracellular glutamate in a 1:1 ratio, restoring redox balance in cells subjected to oxidative stress, amino acid starvation, and metabolic and genotoxic stress (39). Additionally, it is an important antioxidant pathway in ferroptosis. In this system, GPX4 belongs to a family of selenoproteins containing selenocysteine (Sec), which can reduce hydroperoxides, including H_2_O_2_. Humans possess eight isoforms, five of which contain Sec residues (40). GPX4 can reduce phospholipid peroxide to lipohydrol, which is essential for resistance to oxidative stress. The absence of GPX4 renders cells highly sensitive to oxidative stress. Hence, GPX4 activity is essential for ferroptosis triggered by ferroptosis inducers (41). In addition, glutathione (GSH), a tripeptide comprising glutamate, cysteine, and glycine, has important antioxidant functions (42). Intracellularly, GSH, functioning as a cofactor of GPX4, is essential for the antioxidant activity of GPX4 and can bind to free iron, thereby facilitating iron storage and inhibiting ferroptosis (43).GSH, as a major intracellular antioxidant, exists in reduced GSH and oxidized GSSG forms, with the ratio of these forms directly reflecting the redox status of the cell (44). The Xc/GSH/GPX4 system initially facilitates the intracellular transport of cysteine into the cell by the Xc transport system, subsequently enhancing GSH synthesis (45). Subsequently, GPX4 utilizes GSH to mitigate lipid peroxidation by preventing the cascade of lipid peroxidation by reducing phospholipid hydroperoxides to the corresponding alkyl phospholipids, protecting cell membranes from oxidative damage (46).

#### 3.3.2 P53

P53 is a protein that functions as a cellular “guardian” (47). Inside the cell, P53 concentration is typically maintained at a low level to prevent excessive interference with normal cellular activities (48). Upon encountering a crisis, including DNA damage, nutritional deficiencies, insufficient oxygen supply, or free radical attack, the concentration of P53 protein increases rapidly in response to these threats (49). Moreover, P53 is involved in the regulation of the ferroptosis. During ferroptosis, P53 promotes iron endocytosis by regulating long-chain non-coding RNA PVT1 (lncRNA PVT1) to increase the expression of transferrin receptor 1 (TFR1) and by modulating SLC25A28 and ferric reductase (FDXR) to augment iron production (50). Furthermore, P53 stimulates ferroptosis by increasing the expression of glutaminase 2 and ROS production (51). P53 promotes lipid peroxidation and induces ferroptosis by increasing the levels of the SAT1-ALOX15 pathway (52). In addition, P53 can transcriptionally inhibit the expression of the cystine transporter protein SLC7A11, which reduces intracellular cystine uptake, subsequently reduces GSH synthesis, and indirectly inhibits GPX4 enzymatic activities, leading to the accumulation of intracellular lipid peroxides, which promotes ferroptosis. Additionally, p53 can inhibit ferroptosis by promoting its essential target gene p21, thereby inhibiting the cell cycle and diverting some of the raw materials used for synthesizing nucleic acids to synthesize the reducing power NADPH and GSH (53).

#### 3.3.3 Nrf2

Nuclear factor erythroid 2-related factor 2 (Nrf2) is a protein crucial to cellular function. It primarily regulates cellular antioxidant responses to protect cells from damage caused by oxidative stress (54). NRF2, a key regulator in ferroptosis, controls the expression of many antioxidant enzymes and associated annotates through its downstream antioxidant response element (ARE) to maintain intracellular redox homeostasis (55). During ferroptosis, NRF2 activation enhances the antioxidant capacity of cells, inhibits lipid peroxidation, and prevents ferroptosis by upregulating the expression of key enzymes, including GPX4 (56). Furthermore, the activity of NRF2 is inhibited by Keap1; under oxidative stress, NRF2 dissociates from Keap1 and translocates to the nucleus to activate the expression of downstream genes while regulating iron metabolism and affecting iron uptake, storage, and release, thus playing a protective role in ferroptosis (57).

### 3.3.4 Mevalonate pathway

The MVA pathway facilitates the intracellular synthesis of cholesterol and several important non-cholesterol steroids, with 3-hydroxy-3-methyl-glutaryl-coenzyme A reductase (HMG-CoA reductase) serving as the rate-limiting enzyme that regulates cholesterol synthesis in the cell (58). It is essential in GPX4 biosynthesis by providing the isoprenoid moiety necessary for GPX4 synthesis and other selenoproteins (59). When the MVA pathway is inhibited, GPX4 synthesis is compromised, accumulating intracellular lipid peroxides, which may subsequently induce ferroptosis (60). Furthermore, treatment of cells with the ferroptosis inducer FIN56 does not cause GSH depletion; instead, it leads to post-translational GPX4 deletion and a decrease in MVA-derived lipophilic antioxidants (61), indicating that FIN56-induced cellular ferroptosis occurs mainly through MVA pathway modulation.

#### 3.3.5 FSP1-CoQ10-NAD(P)H axis

The mechanism of action of ferroptosis suppressor protein 1 (FSP1) and coenzyme Q10 (CoQ10) in ferroptosis involves a parallel system independent of GPX4 and GSH (62). In this mechanism, FSP1 acts as a flavoproteinase, catalyzing the reduction of coenzyme Q10 from its oxidized form (ubiquinone) to its reduced form (ubiquinol) utilizing NAD(P)H as an electron donor. Panthenol acts as a potent antioxidant that traps and neutralizes lipid peroxyl radicals, resulting in lipid peroxidation and inhibiting ferroptosis (63). In the absence of GPX4 function, FSP1 can independently resist lipid peroxidation and ferroptosis, offering an extra layer of protection for cells (64).

#### 3.3.6 GCH1-BH4 axis

The GCH1-BH4 pathway is an important anti-lipid peroxidation pathway in ferroptosis, which is independent of the conventional Xc-GSH-GPX4 axis and the NADPH-FSP1-CoQ10 pathway (65). GCH1 functions as a rate-limiting enzyme in tetrahydrobiopterin (BH4) synthesis and inhibits ferroptosis through its metabolites BH4 and dihydrobiopterin (BH2) (66). BH4 functions as an antioxidant in the cell and can directly trap free radicals and prevent the overoxidation of phospholipids, which contain two polyunsaturated fatty acid tails, thereby protecting the cell membrane from damage (67). Furthermore, the synthesis and cycling pathways of BH4 are regulated by GCH1 and dihydrofolate reductase (DHFR), respectively, indicating that the metabolic status of BH4 is essential for sustaining cellular redox homeostasis (68). GCH1 overexpression selectively protects membrane phospholipids from peroxidation, and the level of GCH1 expression exhibited a substantial correlation with the sensitivity of cancer cells to ferroptosis (69). BH4 may affect lipid peroxidation-regulated pathways involving CoQ10 by influencing the conversion of phenylalanine to tyrosine, which subsequently interferes with the synthesis of CoQ10 precursors (70).

#### 3.3.7 Antioxidant pathway of peroxiredoxin 6

**Figure 1:**
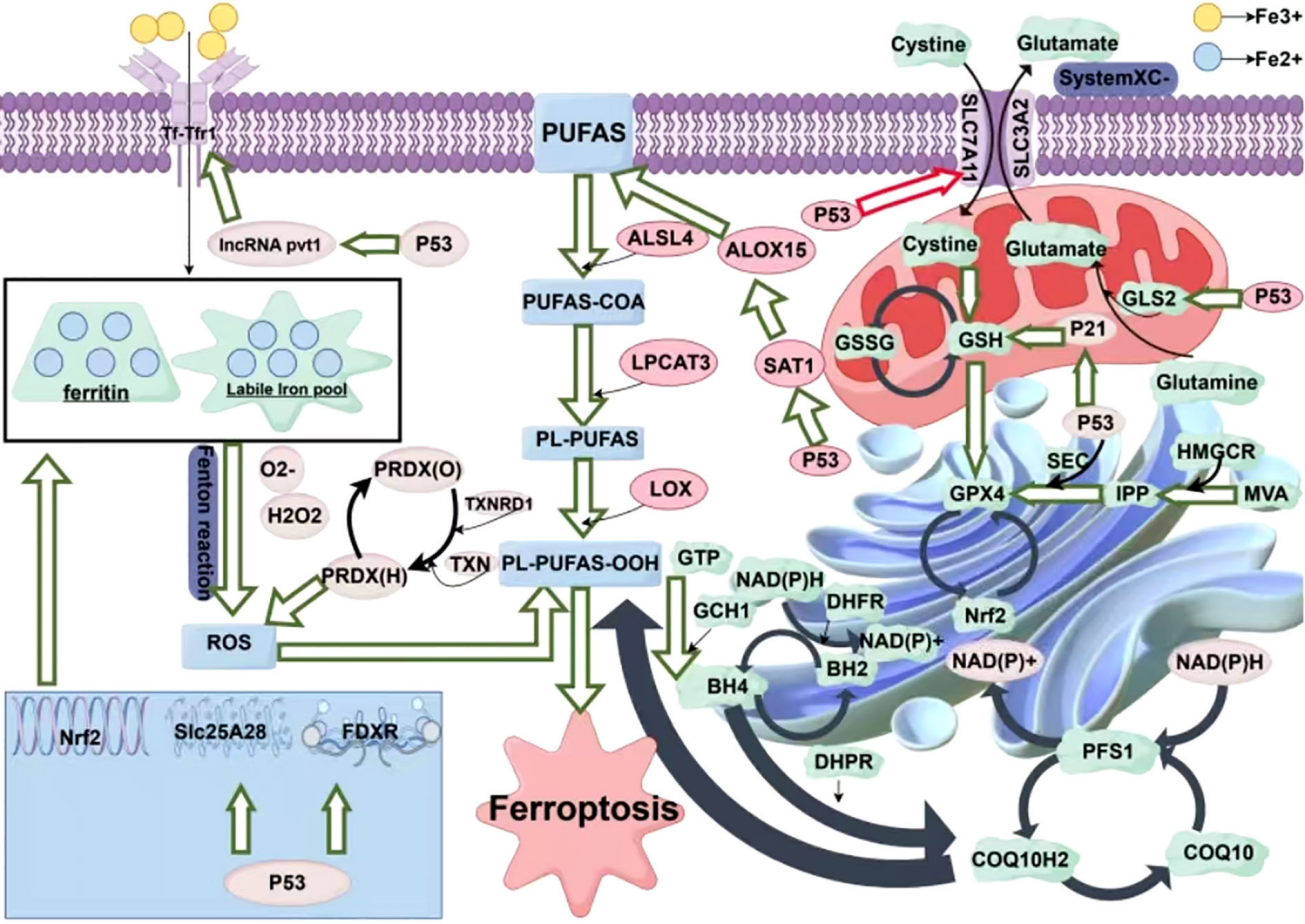
Solute Carrier Family 7 Member 11(SLC7A11); Solute Carrier Family 3 Member 2(SLC3A2); Acyl-CoA Synthetase Long Chain Family Member 4 Gene(ALSL4); Lysophosphatidylcholine Acyltransferase 3(LPCAT3); Tumor Protein P53(P53); Glutaminase 2(GLS2); Glutathione(GSH); Glutathione Disulfide GSSG); Glutathione Peroxidase 4(GPX4); Mevalonate(MVA); 3-Hydroxy-3-Methylglutaryl-CoA Reductase(HMGCR); Isopentenyl Pyrophosphate(IPP); Nuclear Factor Erythroid 2-Related Factor 2 (NRF2); Nicotinamide Adenine Dinucleotide Phosphate(NADPH); Oenzyme Q10(COQ10); Progression-Free Survival 1 (PFS1); Tetrahydrobiopterin(BH4); Polyunsaturated Fatty Acids(PUFAS).

PRDX6 is an enzyme that catalyzes the reduction of H_2_O_2,_ short-chain, and phospholipid hydroperoxides. In the antioxidant defense system, PRDX6 protects cells from oxidative stress-induced damage through its GSH peroxidase and acidic Ca^2+^-independent phospholipase A2 activities (71). PRDX6 negatively regulates ferroptosis in tumor cells. A reduction in PRDX6 expression increases tumor cells’ sensitivity to ferroptosis inducers, indicating that PRDX6 may affect the sensitivity of cells to ferroptosis inducers (72). Besides, PRDX6 deficiency reduces the expression of the intracellular selenoprotein GPX4, which induces ferroptosis. PRDX6 contributes to selenoprotein synthesis by increasing the efficiency of selenium (Se) utilization as a Se-delivering protein, inhibiting ferroptosis (73) (**Figure 1**).

## 4 Correlation between ferroptosis and RA

Recent findings indicate that iron metabolism disorders in ferroptosis, lipid peroxidation, and antioxidant system imbalance are related to the pathomechanism of RA. This indicates a significant association between ferroptosis and RA, implying that inhibiting ferroptosis may mitigate damage to RA.

### 4.1 RA in iron metabolism

Regarding iron metabolism, some studies have demonstrated that disorders of iron metabolism in the body have a strong relationship with the progression of RA disease (74). Hiroe Sato et al. assessed iron metabolism markers, including ferritin, fibroblast growth factor 23, and 25-hydroxyvitamin D, by collecting blood samples of patients with RA for biochemical analysis. They subsequently measured the bone mineral density using dual-energy X-ray absorptiometry. Their statistical analysis revealed that in patients with RA, serum iron tends to correlate positively with ferritin and ferromodulin; however, inflammatory markers correlate negatively with serum iron and positively with ferromodulin (75). In addition, synovial tissue is an important site for RA pathogenesis as well as for the identification of targets (76). A G Mowat’s study demonstrated that iron levels in synovial tissue of patients with RA may be significantly different from those of normal individuals and that the use of medications such as sebaceous steroids in the treatment of patients with RA can inhibit inflammatory activity, while at the same time leading to an increase in the levels of plasma iron and hemoglobin (77). Additionally, animal experiments by A J Dabbagh et al. have demonstrated that intravenous iron administration results in increased synovial inflammation in RA (78). Furthermore, iron is an essential immune response regulator, and its association with iron metabolism has a great impact on autoimmune diseases, including RA, especially in neutrophils, macrophages, and T cells (79). In neutrophils, iron overload can diminish their bactericidal efficacy and disrupt intracellular ROS homeostasis, consequently impairing cellular function. Macrophages may experience impaired function in an iron-overloaded state, including reduced phagocytosis and diminished ferroportin response, limiting their role in immune responses. Moreover, T-cells may experience adverse effects on their activation, proliferation, and cytokine production due to iron overload (80).

### 4.2 RA in lipid peroxidation

Regarding lipid peroxidation, oxidative stress is a major feature of RA, which involves the activation and involvement of various inflammatory cells that release large amounts of ROS and reactive nitrogen species (RNS) in response to inflammation. This process results in the peroxidation of polyunsaturated fatty acids in the cell membranes and the generation of harmful lipid peroxidation products (81). These products can affect the function of T cells, B cells, and macrophages, thereby affecting the immune response in RA. Besides, they can directly affect chondrocytes, promoting the release of cartilage-degrading enzymes, leading to cartilage damage, and stimulating the activation of osteoclasts, which enhances bone resorption and results in bone destruction (82). Shengpeng Zhang et al. reported that the synovial membrane of joints in patients with RA contains lipid peroxidation metabolites that can recognize and bind to cell surface receptors, including toll-like receptor 4 (TLR4), thereby altering the properties of the cell membrane, interfering with cell signaling, and exacerbating the pathological process of RA (83). In patients with RA, high lipid peroxidation levels result in the formation of harmful lipid hydroperoxides and electrophilic reactive lipid compounds, which contribute to an increase in biomarkers of oxidative damage (84). The accumulation of ROS and RNS in cells can activate the transcription factor NF-κB (85). Under normal conditions, NF-κB binds to the inhibitory protein IκB in the cytoplasm (86). Following ROS-induced phosphorylation and degradation of IκB, NF-κB is released and translocated to the nucleus (87). In the nucleus, activated NF-κB binds to the promoter regions of target genes, facilitating the transcription of many inflammatory cytokines, including TNF-α, interleukin 1β (IL-1β), and IL-6 increased expression of these cytokines, leading to amplification of the inflammatory response, as well as the stimulation of synoviocytes proliferation and production of matrix metalloproteinases, thus facilitating inflammation and joint damage in RA (88).

### 4.3 RA in antioxidant system imbalances

Regarding antioxidant system imbalance, Hanzhi Ling et al. reported that the expression level of long-chain specific lipoyl coenzyme A synthetase 4 (ACSL4) was significantly reduced in synovial and fibroblast-like synovial cells (FLS) from patients with RA, while the expression levels of ferritin heavy chain 1 (FTH1), GPX4, and solute carrier family 7 member 11 (SLC7A11) expression levels were comparatively increased (89). Moreover, P53 induces cell cycle arrest and inhibits the proliferation of damaged cells by regulating the expression of cell cycle-related genes, including P21, hence contributing to erythrocyte stability (90).P53 mitigates the symptoms of patients with RA by inhibiting pro-inflammatory factor production, including TNF-α, and by modulating the activation, proliferation, and differentiation of T- and B-cells (91). In the synovium of RA joints, p53 activates apoptosis-promoting pathways, eliminates damaged and inflammatory cells, and reduces inflammatory responses and joint damage (92).

**Figure 2:**
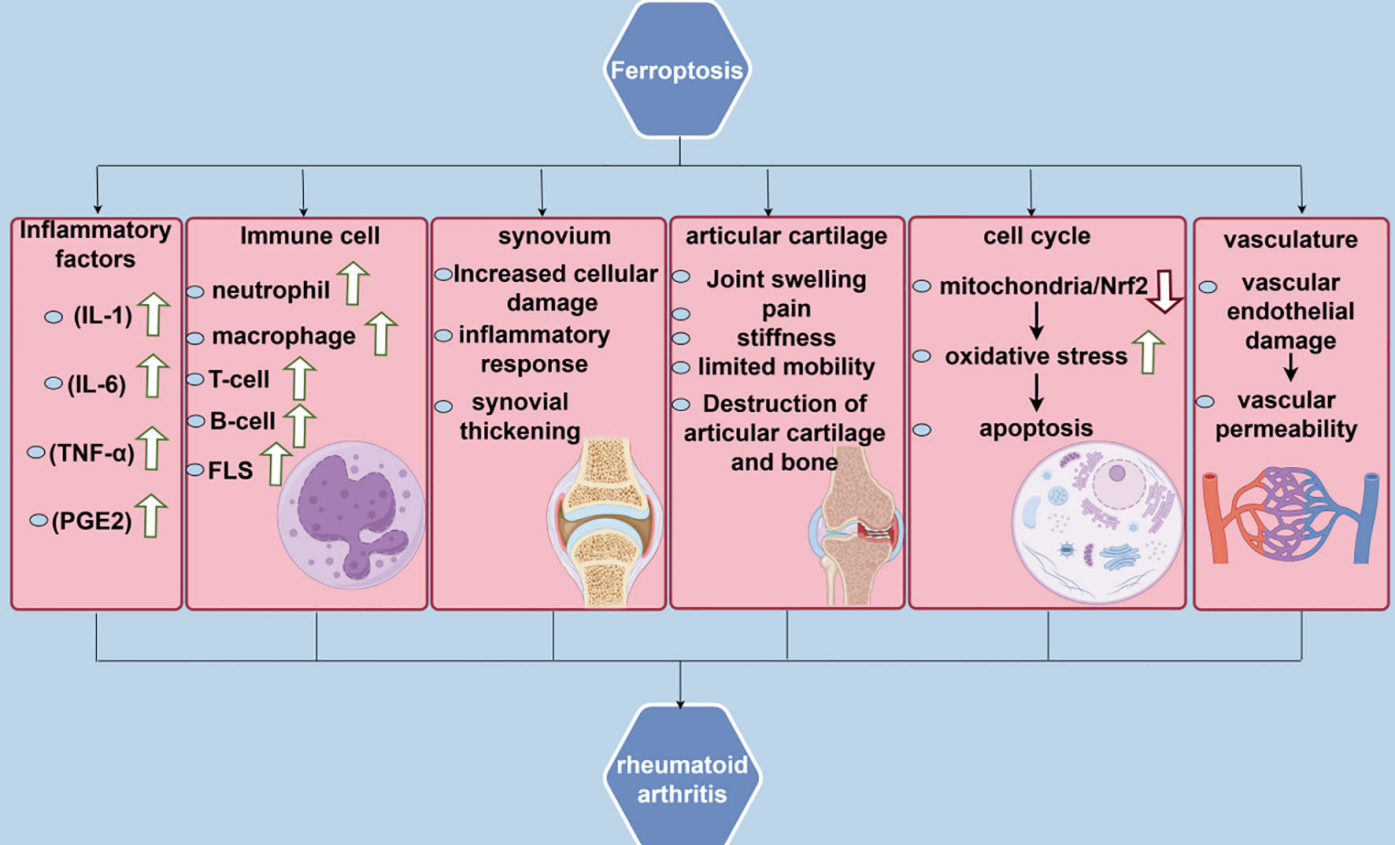
Correlation between ferroptosis and rheumatoid arthritis.

In RA treatment, NRF2 serves as a double-edged sword that scavenges ROS and mitigates oxidative stress damage on joint cells by activating ARE and enhancing the expression of antioxidant enzymes, including GSH peroxidase and superoxide dismutase (93). Meanwhile, NRF2 inhibits the expression of pro-inflammatory factors, including TNF-α and IL-6, thereby exerting an anti-inflammatory effect through the regulation of downstream heme oxygenase-1 (HO-1) and protecting the cells against inflammation-induced damage (94). However, NRF2 activation may affect the efficacy of conventional antirheumatic medications by diminishing their effectiveness through enhanced drug efflux (95). Consequently, careful consideration should be given to the contraindications of dosing NRF2 for RA. Additionally, statins, inhibitors of the MVA pathway, may mitigate inflammation by inhibiting HMG-CoA reductase, thereby decreasing downstream metabolites of the MVA pathway and modulating the immune response (96).There are also some unresolved issues in the study of the antioxidant system in RA.For example, the degree of activation of the Nrf2 signaling pathway in RA and the mechanism of its interaction with other signaling pathways are still not fully understood.In addition, although several studies have shown that there is an imbalance of the antioxidant system in RA patients, there is still a lack of effective strategies and methods on how to precisely regulate the antioxidant system for the purpose of treating RA (**Figure 2**).

## 5 Medications that inhibit ferroptosis in RA treatment

A comprehensive study of the mechanism of ferroptosis increasingly demonstrates that medications that inhibit ferroptosis may have potential applications in RA treatment. These medications inhibit ferroptosis and mitigate the harmful effects of RA by affecting iron metabolism, lipid peroxidation, immuno-inflammatory dysregulation, oxidative stress, GSH depletion, and GPX4 inactivation.

### 5.1 Small molecule medications

#### 5.1.1 Glycyrrhizic acid

GA is the primary bioactive constituent of *Glycyrrhiza glabra*, which possesses numerous bioactivities and neuroprotective effects (97). Xuan Ma et al. reported that GA could inhibit Fin56-induced cellular destabilization by inhibiting iron accumulation and lipid peroxidation while simultaneously increasing the cellular levels of coenzyme Q10. This mechanism mitigates ferroptosis-induced inflammatory injury and protects neurons from damage (98). Yunhui Feng et al. reported that 18β-glycyrrhetinic acid (18β-GA) may inhibit inflammatory cytokine production by inhibiting the MAPK/NF-κB signaling pathway and enhancing FOXO3 signaling, hence reducing synoviocyte proliferation and facilitating apoptosis. Furthermore, 18β-GA mitigated arthropathic changes, reduced serum levels of pro-inflammatory cytokines, and alleviated drug-induced hepatic damage in RA model mice (99).

#### 5.1.2 Cryptotanshinone

CRY exhibits biological activities for the treatment of coronary heart disease, renal failure, Alzheimer’s disease, cancer, obesity, aging, diabetes, and fibrosis of the liver, lungs, heart, and kidneys (100). CRY upregulates GPX4 expression and enhances cellular antioxidant capacity. In addition, CRY inhibits BAX expression (Bcl-2-associated X-protein) and HMGB1 (High Mobility Group Protein 1), thereby reducing iron release, lipid peroxidation, inflammatory response, and cellular damage. Besides, CRY inhibits NF-κB activation and reduces inflammatory factor production, thereby inhibiting ferroptosis (101). In patients with RA, CRY exerts its anti-inflammatory effects primarily by inhibiting the production of inflammatory mediators, modulating the immune response to ameliorate the Th17/Treg cell imbalance, and inhibiting the activation of inflammatory signaling pathways by blocking p300-mediated STAT3 acetylation. Furthermore, CRY reduces joint destruction and protects joint structures (102).

#### 5.1.3 Quercus dentata

Quercetin is a flavonoid that offers many benefits for the human body, including providing cardiovascular protection, anti-inflammatory properties, anticancer activity, anti-ulcer effects, and anti-allergic and antiviral effects (103). In liver injury, it ameliorated non-alcoholic fatty liver disease, diminished triglyceride and cholesterol accumulation, and inhibited mitochondrial ROS production. In the central nervous system, it protects dopaminergic neurons, activates the Nrf2 signaling pathway, and increases the levels of GPX4 and SLC7A11. In BMSC (bone marrow mesenchymal stem cells) injury, it prevents oxidative stress-induced ferroptosis and promotes cell proliferation and osteogenic differentiation. In type 2 diabetes, it decreases iron levels and ferritin levels in pancreatic β-cells and reduces ROS and MDA levels (104). Quercetin inhibits the activation of nuclear factor κB (NF-κB) in RA, thereby diminishing the inflammatory response. In addition, quercetin can activate antioxidant enzymes, including superoxide dismutase (SOD), GPx, and HO-1, which enhances the cellular antioxidant defense system (105).

#### 5.1.4 Epigallocatechin gallate

EGCG is a polyphenolic compound in green tea that possesses many biological activities, including antioxidant, anti-inflammatory, antitumor, antimetabolic disease, cardiovascular, and neuroprotective effects (106). Lin Yue et al. reported that EGCG can enhance the antioxidant capacity of the cells by activating the Nrf2/HO-1 signaling pathway, mitigating oxidative stress during ferroptosis. Ming-Shan Chen et al. reported that adipose tissue-derived mesenchymal stem cells (ADSCs) exhibited neuroprotective and anti-inflammatory functions in patients with RA, while EGCG-pretreated ADSCs demonstrated significantly enhanced neuroprotection and were able to reactivate RA-induced inhibition of the PI3K/Akt survival pathway (107), thereby inhibiting RA progression.

#### 5.1.5 Artemisinin

ATT, a sesquiterpene lactone extracted from the Chinese herb *Artemisia annua* with a specific peroxyl group, is a first-line drug for malaria treatment globally (108). Peng-Xi Deng et al. reported that Nrf2 can be activated and translocated to the nucleus in the presence of ATT, subsequently binds to the ARE, which upregulates the expression of various antioxidant genes and enhances GSH synthesis, thereby inhibiting ferroptosis (109). Additionally, ATT increased the expression and activity of GPX4 through the Nrf2-SLC7A11-GPX4 pathway, thereby diminishing intracellular phospholipid peroxide accumulation, preserving cell membrane integrity, reducing oxidative stress, and preventing Erastin-induced cell death (110). In RA treatment, Jian Chen et al. identified intercellular adhesion molecule 2 (ICAM2) as a key factor promoting RA progression in RA-FLS by RNA sequencing analysis. ATT inhibited RA progression by inhibiting the ICAM2/PI3K/AKT/p300 pathway in RA-FLS and inhibited METTL3-mediated ICAM2N6-methyladenosine modification of mRNA. Furthermore, p300 directly promotes METTL3 transcription, which can be inhibited by ATT in RA-FLS. Expressions of METTL3, ICAM2, and p300 in synovial tissues of patients with RA are correlated with clinical features and response to therapy, serving as potential biomarkers of therapy response (111).

#### 5.1.6 Resveratrol

Res is a non-flavonoid polyphenolic compound and an antitoxin secreted by plants in response to stressors or pathogenic threats, with synthesis significantly increased, especially in response to UV irradiation, mechanical injury, and fungal infections (112). In a heart failure model, resveratrol mitigates ferroptosis by decreasing p53 K382 acetylation levels, reducing SLC7A11 degradation, and increasing cellular concentrations of GSH and GPX4. Furthermore, in the lipopolysaccharide (LPS)-induced ferroptosis model of HT-29 cells, resveratrol downregulated the expression of pro-oxidant indicators, inflammatory factors, total cellular ferric ions, and the negatively regulated ferroptosis gene, *GPX4*, while upregulating the expression of the positively regulated factor for ferroptosis, ACSL4, and the pathway indicators, SIRT1 and NRF2, leading to ferroptosis inhibition (113). Additionally, resveratrol exerts an inhibitory effect on inflammation-associated signaling pathways, including NF-κB, hence diminishing inflammation-mediated ROS generation. Besides, it inhibits ROS production by activating the SIRT1-Nrf2 signaling pathway, thereby reducing RA prevalence (114).

#### 5.1.7 Apigenin

Apigenin is a flavonoid prevalent in fruits and vegetables and is recognized for its several health benefits, including antitumor, cardiovascular protection, anti-neurodegenerative diseases, and anti-type 2 diabetes (115). Apigenin upregulates the expression of antioxidant enzymes and downregulates pro-inflammatory factors through activation of the AMPK/Nrf2/HO-1 signaling pathway. It inhibits oxidative stress, reduces reactive oxygen species generation, and enhances intracellular antioxidant capacity, resulting in significant inhibition of ferroptosis at the cellular level (116). Monu et al. reported that apigenin can regulate transthyretin (TTR) by modulating TNF-α-stimulated expression of TTR and receptor for advanced glycosylation end-products, thereby attenuating the inflammatory response and cartilage destruction in RA. Furthermore, silymarin exerts anti-inflammatory effects in patients with RA by reducing the levels of p65 (a key component of the NF-kB pathway), inhibiting NF-kB pathway activation, and reducing inflammatory factors production (117).

#### 5.1.8 Silymarin

Silymarin is an antioxidant extracted from the milk thistle plant. Silymarin can inhibit iron autophagy by directly targeting FTH1 and consequently affecting the NCOA4-FTH1 interaction in acute kidney injury mouse models (118). In patients with RA, silymarin exerts anti-inflammatory effects by inhibiting the NF-κB pathway and diminishing the production of pro-inflammatory cytokines IL-6 and IL-1β in RA-FLS. *In vitro*, silymarin inhibits Th17 cell differentiation and reduces IL-17 and RORγ expression, thereby alleviating RA symptoms (119).

#### 5.1.9 β-Stilbene

Beta-caryophyllene (BCP) is a naturally occurring chemical compound, especially in the essential oils of many plants, and is often used as a natural flavoring agent for foods (120). Yan-Ting Wu et al. reported that BCP inhibits ferroptosis and reduces the expression of ferroptosis-associated inflammatory factors by inhibiting lipid peroxidation in macrophages through activation of the type 2 cannabinoid receptor (CB2R). In addition, BCP diminished the mRNA expression of Tnf-α and Ptgs2 and blocked the activation of MAPK and NF-κB signaling pathways, which significantly inhibited ferroptosis-induced inflammatory responses (121). BCP effectively treats RA by inhibiting intracellular signaling pathways, including the Janus kinase/signal transducer and activator of transcription (JAK/STAT) pathway or the nuclear factor-κB (NF-κB) pathway, thereby diminishing the formation of pro-inflammatory cytokines during inflammation and attenuating the inflammatory response. Furthermore, BCP effectively inhibits the activation of the NLRP3 inflammasome and reduces pro-inflammatory cytokine IL-1β production, thereby reducing inflammatory symptoms in patients with RA (122).

#### 5.1.10 Vitamin E

Vitamin E is a class of fat-soluble compounds that are antioxidants and perform various physiological functions in the body, including safeguarding cell membranes from free radical damage, bolstering the immune system, facilitating blood coagulation, and aiding the nervous system and muscle function (123). It significantly contributes to ferroptosis by inhibiting the Fenton reaction involving iron ions (particularly Fe^2+^) by binding to them, thereby reducing the generation of ROS (124). Moreover, vitamin E maintains GPX4 activity and synergizes with other antioxidants (vitamin C and coenzyme Q10) to prevent lipid peroxidation and inhibit ferroptosis (125). However, in patients with RA, vitamin E reduces the synthesis of inflammatory mediators, including TNF-α and ILs, thereby reducing the inflammatory response (126). It may affect immune cell activation and function, including T and B cells, regulating the immune response and reducing autoimmune attacks, or by affecting intracellular signaling pathways, including nuclear factor-κB (NF-κB) and mitogen-activated protein kinases (MAPKs), which subsequently affect inflammatory and immune responses (127). In addition, some drugs, including methotrexate (MTX), may cause hepatotoxicity in RA treatment, and vitamin E may help minimize the side effects of these drugs (128).

#### 5.1.11 Ginkgolide B

Ginkgo biloba is an ancient tree species of significant medicinal potential, with its leaves and seeds containing abundant biologically active components for treating cardiovascular and cerebrovascular diseases (129). Jing Chen et al. reported that GB can mitigate lipid metabolism disorders by lowering ROS levels and diminishing the accumulation of lipid ROS in cell membranes by inhibiting GPX4 ubiquitination, thereby maintaining its antioxidant properties and inhibiting ferroptosis (130). GB successfully diminished pro-inflammatory cytokines in the sera of patients with RA, including IL-1, IL-6, and TNF-α, thereby inhibiting the activation of inflammatory cells and the release of inflammatory mediators and thus mitigating the inflammatory response in patients with RA. Additionally, GB effectively induced apoptosis in RA FLS, which contributed to the attenuation of synoviocytes, thereby slowing down the pathological progression of RA (131).

#### 5.1.12 Lipistatin

Lipstatin is a secondary metabolite synthesized by streptomyces toxins (132). Hong-Fa Yan et al. reported that lipstatin decreased intracellular iron ion levels and mitigated the Fenton reaction involving iron ion levels, thus reducing ROS production and maintaining GPX4 activity. Furthermore, lipstatin inhibited the TLR4/MyD88/NF-κB signaling pathway, leading to the transformation of the immune response into an anti-inflammatory response. Statins can inhibit the NF-κB pathway and exert their anti-inflammatory effects, thereby reducing the inflammatory response in patients with RA (133).

#### 5.1.13 Celastrol

Celastrol, a naturally occurring compound extracted from the thunder god vine (Tripterygium wilfordii Hook F), has been extensively studied for its anti-inflammatory, antioxidant, and immunomodulatory activities (134). Minling Pan et al. reported that tretinoin inhibits ferroptosis and protects cells from damage through multiple mechanisms, including activation of the AKT/GSK3β signaling pathway, the reduction of lipid peroxidation levels and mitochondrial ROS, and attenuation of ferroptosis. Additionally, tretinoin modulates intracellular iron metabolism and antioxidant responses by upregulating the expression of heme oxygenase 1 (HO-1), further inhibiting ferroptosis (135). Tretinoin, as a potential therapeutic agent for RA, reduces angiogenesis and promotes FLS apoptosis by inhibiting vascular endothelial growth factor, which slows down the progression of the disease and reduces synovial hyperplasia (136). Moreover, tretinoin can reduce joint inflammation by inhibiting Ca^2+^/calmodulin-dependent kinase kinase-β-AMP-activated protein kinase-mTOR pathway and the PI3K/AKT/mTOR signaling pathway and interfere with the COMMD protein interactions to inhibit synovial B-cell infiltration and exert immunosuppressive effects (137).

### 5.2 Nanomaterials

Se possesses beneficial antioxidant qualities and is an essential trace element for health maintenance; it is specifically due to these antioxidant properties that Se nanoparticles can also be used for ferroptosis inhibition (138). For instance, SeNPs activated the Nrf2 signaling pathway and enhanced the expression of antioxidant genes, including GST, SOD, and GPX, thereby enhancing the antioxidant capacity of cells (139). SeNPs effectively upregulated the expression of antioxidant genes associated with ferroptosis, including GPX4, SLC7A11 (cystine/glutamate transporter), FTH1 (ferritin heavy chain), and Fpn1 (iron transporter protein 1), thereby inhibiting ferroptosis (140). In addition, SeNPs can regulate iron metabolism and reduce the accumulation of intracellular iron ions, thereby reducing the risk of ferroptosis (141). In RA models, SeNPs exhibited anti-inflammatory effects by increasing the activity of antioxidant enzymes and improving the redox state of the inflamed synovium. Furthermore, SeNPs exhibited free radical scavenging activity *in vivo*, which helps reduce oxidative stress and thereby alleviate RA symptoms (142).

Tetrahedral framework nucleic acid (tFNA) is a new nanomaterial (143). tFNA, as a novel nanoparticle, was found by Lu Tan et al. to inhibit ferroptosis by promoting cell viability, reducing iron levels 2+, lipid peroxidation, MDA, and LDH, and increasing GSH levels in Aβ-treated N2a cells (144). Additionally, RNA sequencing in the experiment demonstrated that tFNA could counteract the stimulatory effect of Aβ on the ferroptosis driver gene Atf3 and the inhibitory effect on the ferroptosis repressor *Rrm2* and *Furin* genes (145). Simultaneously, tFNA has demonstrated considerable potential in RA treatment. Some medications for RA treatment, including poorly water-soluble or easily degradable medications, can be encapsulated or conjugated by tFNA to improve their stability and bioavailability. For instance, curcumin (Cur) is a natural compound with anti-inflammatory effects; however, its rapid degradation and poor water solubility impede therapeutic efficacy (146). The therapeutic efficacy of Cur can be enhanced by tFNA delivery. Besides, tFNA can transport anti-inflammatory molecules, including miRNAs, to regulate the inflammatory response. For instance, the integration of miR-23b into tFNA enhances its stability, facilitating effective delivery into organisms to inhibit synovial inflammation and cartilage matrix degradation (147).

Liposomes are biocompatible nanovesicles composed of natural phospholipids that protect medications, regulate targeted delivery, control the release rate, and can be enhanced through surface modifications (148). Rosmarinic acid (RosA) encapsulated in nanoliposomes can be prepared into a new delivery system, liposomes with rosmarinic acid (RosA-LIP). This rosmarinic acid liposome (RosA-LIP) leverages the anti-inflammatory and antioxidant properties of rosmarinic acid to reduce cellular iron uptake by inhibiting the expression of TfR1 in cerebral microvascular endothelial cells, thus inhibiting ferroptosis (149). As for RA treatment, GSH was sequestered in liposomes using methods including film dispersion, and liposomal GSH significantly reduced RF, MDA, and CRP levels but demonstrated its antioxidant and anti-inflammatory effects. GSH itself is a potent antioxidant, and liposomal encapsulation improves its stability and bioavailability, allowing it to reach the site of inflammation more effectively. Additionally, liposomal GSH was able to reduce the levels of the pro-inflammatory marker CRP, indicating its anti-inflammatory and joint inflammation-reducing effects, reducing joint damage and pathology scores (150).

### 5.3 Others

#### 5.3.1 Hypoxia-inducible factor 1α subunit

HIF-1α is a transcription factor that activates the expression of several genes involved in cellular adaptive responses to hypoxia, including angiogenesis, energy metabolism, cell survival, and apoptosis. HIF-1α works by inducing the expression of antioxidant enzymes, including GPX and SOD, which help to scavenge ROS that reduce oxidative stress, thereby inhibiting ferroptosis (151). In addition, HIF-1α increases the expression of iron storage proteins, including ferritin, and reduces the availability of free iron ions in the cell, thereby reducing ferroptosis (152). HIF-1α is essential in RA treatment by regulating joint angiogenesis, participating in the inflammatory response, being induced to be expressed by pro-inflammatory cytokines, including IL-1, and modulating the course of inflammation through multiple signaling pathways, including the PHDs/HIF-1α/pVHL pathway and the inhibitor HIF (153).

#### 5.3.2 Coenzyme Q10

CoQ10 is a prominent antioxidant that binds to ferroptosis inhibitory protein 1 (FSP1) to form the FSP1/CoQ10 system, collaboratively inhibiting ferroptosis (154). J Kucharská et al. reported that CoQ10 treatment of arthritic rats for 28 days slightly reduced inflammatory markers and increased plasma antioxidant capacity. Furthermore, CoQ10 can exert an anti-inflammatory effect by reducing the production of inflammatory mediators, including TNF-α and IL-1β (155).

In summary, iron death has an important role in the pathogenesis of RA, and a variety of iron death inhibitors show great promise in the treatment of RA.However, there are many challenges in the current study and differences in efficacy, translational potential and challenges among different inhibitors.In terms of small molecule drugs, although several drugs have shown promising efficacy *in vitro* and in animal models, their data in clinical studies are still limited, and long-term efficacy and safety need to be further validated. In addition, the issue of selectivity and potential toxicities of small molecule drugs are also challenges.For example, resveratrol, despite its multiple health benefits, has a relatively weak specific inhibitory effect on iron death and may act through multiple signaling pathways, which may lead to interference with its effects in RA treatment by other biological processes (156). In contrast, compounds such as mannuronic acid may have greater specificity, but their efficacy may be influenced by cell type and pathological environment (157). Nanomaterials, as a novel therapeutic tool, have the advantages of strong targeting, high bioavailability and delivery efficiency, but still face challenges in their preparation process, clinical translation and biocompatibility (158). For example, potential issues such as the toxicity of selenium nanoparticles and the immunogenicity of liposomes need to be further investigated and addressed.Future studies should focus more on the in-depth evaluation and optimization of iron death inhibitors to improve their efficacy and safety and accelerate their translational application in RA therapy.In addition, there are significant differences in specificity and off-target effects among different compounds.These differences suggest that we need to comprehensively consider the specificity, efficacy, and potential off-target effects when developing and selecting iron death inhibitors to optimize therapeutic strategies (**Table 1**).

**Table 1:** Drugs that inhibit ferroptosis in the treatment of RA.

Categorization	Veterinary drug	Acts on Ferroptosiss	Role in RA	Test model
Small molecule drug	Glycyrrhizic acid	Inhibits cellular destabilizing iron accumulation and lipid peroxidation, increases cellular levels of coenzyme Q10	Inhibits the production of inflammatory cytokines, decreases synovial cell proliferation, and promotes apoptosis	LPS or TNFα-induced inflammatory cell model and collagen-induced arthritis (CIA) animal model
	Cryptotanshinone	Upregulates GPX4 expression, inhibits BAX and HMGB1 expression, and suppresses NF-κB activation.	Inhibition of inflammatory mediators, modulation of immune response to improve Th17/Treg cell imbalance, blocking p300-mediated STAT3 acetylation, and reduction of joint destruction	Establishment of PCOS rat model by daily injection of human chorionic gonadotropin and insulin for 22 days
	Quercetin	Inhibition of ROS production, activation of the Nrf2 signaling pathway, increased GPX4 and SLC7A11 levels, etc.	Inhibition of nuclear factor-κB (NF-κB) activation and activation of antioxidant enzymes such as superoxide dismutase (SOD), glutathione peroxidase (GPx) and heme oxygenase-1 (HO-1)	Lipopolysaccharide (LPS)/Ovalbumin (OVA)-induced neutrophil asthma mouse model
	Epigallocatechin gallate	Activation of the Nrf2/HO-1 signaling pathway, interaction with KEAP1 protein, and promotion of Nrf2 release from KEAP1 inhibition	Can reactivate RA-induced inhibition of the PI3K/Akt survival pathway	Acute Kidney Injury (AKI) Model Induced by Gentamicin (GEN)
	Arteannuin (anti-malaria chemical)	Increased GSH synthesis and the Nrf2-SLC7A11-GPX4 pathway increased GPX4 expression and activity	Inhibition of RA progression by inhibiting ICAM2/PI3K/AKT/p300 pathway in RA-FLS	Hippocampal HT22 cell model
	Resveratrol	Low p53 K382 acetylation levels, reduced SLC7A11 degradation, and increased cellular levels of GSH and GPX4	Inactivates reactive oxygen species and activates the SIRT1-Nrf2 signaling pathway to inhibit ROS production.	Lipopolysaccharide (LPS)-induced Ferroptosis in HT-29 cells, a model of heart failure
	Apigenin	Activation of the AMPK/Nrf2/HO-1 signaling pathway	Regulation of TNF-α-stimulated TTR and RAGE expression and inhibition of NF-kB pathway activation	Mouse splenocytes and S180 cells
	Thistlebutin	Inhibition of the activity of Ferroptosis inducers such as RSL3 and erastin	Inhibition of NF-κB pathway reduces production of pro-inflammatory cytokines IL-6 and IL-1β in RA-FLS	Acute kidney injury model, two-month-old C57 BL/6J wild-type mice
	β-Caryophyllene oxide	Inhibition of lipid peroxidation in macrophages blocked activation of MAPK and NF-κB signaling pathways	Inhibits intracellular signaling pathways and reduces production of the pro-inflammatory cytokine IL-1β	A mouse model of DSS-induced colitis
	Ginkgolide B	Reduces the accumulation of lipid reactive oxygen species in cell membranes and decreases intracellular iron content	Reduction of pro-inflammatory cytokines in the serum of RA patients and promotion of apoptosis in RA fibroblast-like synoviocytes (FLS)	C57BL/KsJ db/db mice fed a high-fat, high-sugar diet
	Vitamin E	Fenton reaction involving iron ions to prevent lipid peroxidation	Reduces the production of inflammatory mediators, affects the activation and function of immune cells, and influences intracellular signaling pathways	A model of pentylenetetrazol-induced epilepsy
	Lipstatin	Reduces intracellular iron ion levels, decreases ROS production and maintains GPX4 activity	Inhibition of TLR4/MyD88/NF-κB signaling pathway, inhibition of NF-κB pathway	Auranofin drug-induced Ferroptosis in mice
	Celastrol	Reduced lipid peroxidation levels and ROS, inhibited PRDXS antioxidant activity, and upregulated (HO-1) expression	Reduces angiogenesis, promotes synovial fibroblast (FLS) apoptosis, reduces synovial hyperplasia, and inhibits synovial B-cell infiltration	Cisplatin-induced acute kidney injury (CI-AKI) mouse model
Nanomedicine	Selenium nanoparticles (SeNPs)	Improvement of cellular antioxidant capacity, up-regulation of the expression of anti-Ferroptosis genes, and reduction of intracellular iron ion accumulation.	Enhances antioxidant enzyme activity, free radical scavenging activity	SeNPs-AOS in attenuating heat stress-induced oxidative damage in broiler organ models
	Tetrahedral framework nucleic acid (tFNA)	Increase in GSH levels, reversal of the promoting effect of the Ferroptosis driver gene Atf3, and inhibition of the Ferroptosis repressors Rrm2 and Furin genes	Improved drug stability and bioavailability carrying anti-inflammatory molecules	APP/PS1 double transgenic mouse model
	liposome (bilayer lipid vesicle)	Liposomes of rosemarinic acid inhibit Ferroptosis by inhibiting TfR1 expression in brain microvascular endothelial cells and reducing cellular iron uptake	Liposomal glutathione reduces rheumatoid factor, malondialdehyde and C-reactive protein levels, decreases levels of pro-inflammatory marker CRP, anti-inflammatory and reduces joint inflammation and joint damage	niosomes (vesicles composed of nonionic surfactants) and liposomes (vesicles composed of phospholipids)
Other	Hypoxia-inducible factor 1α subunit (HIF-1α)	Induction of antioxidant enzymes and increased expression of iron storage proteins such as ferritin	Regulates angiogenesis in the joints, participates in the inflammatory response, is induced to express by pro-inflammatory cytokines such as IL-1, and through multiple signaling pathways	A model of Ferroptosis in adipose tissue of ketotic cattle
	Coenzyme Q10	Binds to (FSP1) to form the FSP1/CoQ10 system to jointly inhibit Ferroptosis	Reduced production of inflammatory mediators	CT26 bilateral tumor model

## 6 Conclusion and outlook

RA treatment is advancing. However, it faces some challenges. Recently, the therapeutic approach for RA has gradually transitioned from pure symptom relief to early intervention and a (treat-to-target) strategy, which involves continual assessment and modification of the treatment regimen within six months of initiation, guided by setting clear treatment goals (clinical remission or low disease activity). This strategy has significantly improved patient prognosis and reduced joint damage and disability. However, challenges persist in RA treatment, including the poor response of some patients to the available medications and the long-term safety and drug resistance of biologics that need to be further studied. Furthermore, there is still room for improvement in the treatment compliance rate of patients with RA in China, and multidisciplinary comprehensive management and individualized treatment need to be strengthened (159). As a new form of apoptosis, ferroptosis has gradually gained attention in the study of RA in recent years. However, ferroptosis, which is characterized by iron-dependent lipid peroxidation and excessive accumulation of ROS, may exacerbate the disease progression by facilitating the activation of inflammatory cells and causing oxidative damage to joint tissues in patients with RA. For instance, most of the chemical compounds mentioned in this study mostly reduce inflammation and immunity in RA while inhibiting ferroptosis. Accordingly, we believe that inhibiting ferroptosis may provide new therapeutic options for RA, especially in regulating iron metabolism and antioxidant defense systems [157]. Despite the understanding of the role of iron death in RA, there are still many contradictions and limitations in existing studies.For example, there is current controversy about the specific role of iron death in different pathogenic stages of RA, with some studies pointing to a possible potential protective role in the early stages of the disease, while others emphasize its pro-inflammatory effects in disease progression.In addition, there is a lack of clarity regarding the interactions between iron death and other modes of cell death (e.g., apoptosis, necrosis, pyroptosis, etc.) in RA; for example, there may be a cross-regulatory mechanism between iron death and apoptosis, and the two may be both independent of each other and interact with each other in the pathologic process of RA.In some cases, iron death may promote an inflammatory response through the release of proinflammatory factors, while dysregulation of apoptosis may also exacerbate iron death.This complex interaction may lead to different cell death patterns exhibited at different pathological stages of RA, thereby affecting disease progression and therapeutic response (160). However, care should be taken when promoting ferroptosis in target cells to inhibit excessive death in other cells to prevent other damage (79).

The study of ferroptosis and RA should be advanced further to clarify the relationship between ferroptosis and RA, particularly regarding how iron metabolic imbalance affects inflammatory response, joint damage, and other mechanisms. However, developing ferroptosis inhibitors will become a research focus. Currently, most medications administered in clinical settings have significant side effects and poor water solubility after administering the drugs, resulting in decreased efficacy. Consequently, researchers are focusing on the advancement of delivery technologies that can overcome biological barriers, enhance drug efficacy, and minimize side effects. This includes the investigation of nanomedicines that work more precisely at the target site and natural drugs with lower resistance and side effects to achieve a more effective and efficient delivery of ferroptosis drugs. Besides, natural drugs with lower drug resistance and side effects achieve more efficacious RA treatments. Additionally, future gene therapy for RA needs to be focused on ferroptosis, with multi-targeted interventions to reshape the redox balance and inflammatory microenvironment. We can focus on developing intelligent delivery systems, investigating epigenetic-metabolic interaction networks, and establishing biomarker-based precision therapeutic systems. With the ongoing advancement in CRISPR technology and delivery vectors, ferroptosis-targeted gene therapy is expected to be a revolutionary strategy for RA treatment (161). The main mechanism of most current ferroptosis inhibitors involves the inhibition of the Nrf2 pathway and the inhibition of GPX4 and ROS generation. Further insights into the mechanisms and the effects of other ferroptosis pathways in relation to RA are needed.
